# DArTseq Analysis of Cypriot Common Bean Germplasm Unveils an Assortment of Unexplored Genetic Variability

**DOI:** 10.3390/plants14193000

**Published:** 2025-09-28

**Authors:** Maria-Dimitra Tsolakidou, Angelos C. Kyratzis, Nikolaos Nikoloudakis

**Affiliations:** 1 Department of Agricultural Science, Biotechnology and Food Science, Cyprus University of Technology, Limassol 3036, Cyprus; maria.tsolakidou@cut.ac.cy; 2 Vegetable Crop Sector, Agricultural Research Institute, Ministry of Agriculture, Rural Development and Environment, Nicosia 1516, Cyprus; akyratzis@ari.moa.gov.cy

**Keywords:** heirloom varieties, landraces, linkage disequilibrium, *Phaseolus vulgaris*, SNPs

## Abstract

The common bean (*Phaseolus vulgaris* L.) is a globally significant crop with a well-documented domestication history and a critical role in food security. Here, we present the first whole-genome genetic characterization of Cypriot common bean landraces and heirloom varieties, originated from remote mountainous areas, using DArTseq-based SNP genotyping. A total of 13,215 high-quality SNPs were investigated from 50 genotypes, indicating a moderate linkage disequilibrium, high incidences of private and fixed alleles, and an overall low heterozygosity. The comparison of varieties indicated that dry and green bean varieties consisted of genetically distinct clusters, reinforced by phylogenetic and Bayesian structure analyses. A few of the varieties, such as “Gliastro” and “Stringless Blue Lake,” demonstrated an intense genetic diversity and/or inbreeding, whereas others showed evidence of admixture. The outcomes highlight the unique genetic make-up of the Cypriot bean germplasm and its worth as a tool for breeding, conservation, and upcoming genomic-assisted improvement programs.

## 1. Introduction

The common bean (*Phaseolus vulgaris* L.) is globally recognized as a cornerstone staple crop with a millennia-long contribution to the human diet and a remarkable diversity of types, cultivation schemes, and consumption patterns. In parallel, the adaptability of beans to diverse edaphoclimatic conditions, coupled with their high nutritional value (rich in protein and vitamins), has established them as a crucial crop for food security and food sovereignty, especially in developing countries [1]. A rise in bean demand during the last three decades has upped its production (60% increase) and harvested area (more than 30%), as recently reported (FAO, 2023). In terms of worldwide diffusion, the dry bean yield and harvested area (2023) were estimated to be around 28.7 million metric tons/38.4 million hectares (dry beans) and 1.4 million metric tons/134.2 thousand hectares (string beans), respectively. Nowadays, Asia (mainly India and China) is the continent that produces the most green and dried beans, accounting for 91.9% and 49.3% of global output, respectively [2]. India is leading the global production of these legumes (dry or string beans), which accounts for more than 43% of the total output.

Despite current trends, the common bean’s evolutionary drive began in the Americas, where independent domestication events shaped its genetic diversity. Specifically, *P. vulgaris* underwent domestication in two distinct regions: Mesoamerica and the mountainous Andes [3,4]. The beans’ cultivation was later introduced to the Old World (Europe) via conquistadors following Columbus’s voyaging explorations [5]. Germplasm dissemination has probably followed several rounds of introduction from both the Andean as well as the Mesoamerican germplasm, while local factors have formed heterologous populations (landraces) with distinct characteristics [3]. However, the genetic landscape of beans in Europe is not entirely defined by these initial introductions. The subsequent hybridization, adaptation, and gene flow among the Andean and Mesoamerican gene pools have profoundly shaped the genetic structure of European common bean populations, creating heirloom varieties [6].

This centuries-long presence in Europe has created a secondary tailormade domestication effect, resulting in a unique genetic, phenotyping, and agronomic diversity [7,8,9]. Across the several conclusions that were outlined from research focusing on the European germplasm, a key finding is the consistent observation of genotype-by-environment interactions, highlighting the adaptive plasticity of these landraces and their ability to thrive in diverse and/or specific environments. As a result, the conservation of bean landraces is of paramount importance in order to maintain biodiversity, and at the same time ensure future food security, since these gene pools represent a vast reservoir of genetic diversity, harboring valuable traits not found in modern cultivars [10,11,12]. In addition, such lines can further be used via Genomics-assisted breeding (GAB) and the utilization of landraces’ desirable genes [13,14]. In that sense, studies that genetically characterize the *Phaseolus* spp. germplasm align with that scope.

Several marker systems have been used until now to decipher the genomic complexity of beans. Among these, studies employing AFLPs [15,16], ISSRs [17], RAPD [18,19], RFLPs [20], and SSRs [21,22] stand out. Notwithstanding, many of these marker systems encompass several caveats, such as low density, genomic coverage, repeatability, or the inability to detect heterozygous genotypes. Moreover, the information-to-cost index is quite low in contrast to novel genotyping technologies such as Next Generation Sequencing (NGS), which nonetheless pose a significant cost for large-scale analyses [23].

Nowadays, many scientific efforts focus on DArTseq as an economic yet powerful marker analysis employing thousands of SNPs. DArTseq is characterized as an inexpensive, straightforward, and efficient genotyping-by-sequencing method, allowing for a genome-wide marker identification through a restriction enzyme-mediated genome complexity reduction and sequencing of the restriction fragments [24]. This procedure has generated significant insights for the common bean germplasm across countries like Brazil [25], Colombia [26], Kenya [27], Uganda [28], Ethiopia [29], Croacia [30], Turkey [31,32], and Zimbabwe [33].

Cyprus, the third largest Mediterranean island, has a relatively high floristic diversity, and it is considered as a biodiversity hotspot. This is mainly the result of its geographical position at the crossroads of the Eurasian and African continents, its size (9251 km^2^), varied geology and geomorphology, climatic conditions, habitat diversity, long history of human presence, and geographic isolation [34]. For these reasons, several studies focusing on the unique genetic distinctiveness of Cypriot landraces and local groups across crops have provided significant insights into their unique properties and exploitations [35,36,37,38,39].

For centuries, legumes, including common beans, have been appraised as a stable food in Cyprus. At the beginning of the previous century, common beans were mainly cultivated in the mountainous areas of Troodos (Marathasa and Pitsilia region) and to a lesser extent in plains [40]. Between 1930 and 1950, a rapid expansion of the cultivation area was recorded [41] reaching up to 1750 ha and ranking as the 2nd most important legume after broad beans (*Vicia faba*). The two major cultivation centers were Troodos massif (summer crop) and Morphou plain (winter and summer crop). Several local landraces have been cultivated by farmers, with a distinction of those adapted to high-altitude mountainous regions and those adapted to low-altitude plains [42]. Nowadays, these landraces are severely threatened with extinction due to their replacement with modern varieties in the plains, the aging of the rural population, and the abandonment of the agricultural land in the mountainous areas.

The current work aims to characterize the local Cypriot common bean landraces and heirloom variety collection originating from remote mountainous areas by employing a DArTseq analysis. This will enable us to recognize the origin and genetic diversity of the indigenous bean germplasm, while creating guidelines/datasets to mitigate genetic erosion. Finally, based on the SNP analysis among dry and green bean genotypes, possible cross-hybridizations among the two lineages may be identified, aiding future breeding/conservation efforts.

## 2. Materials and Methods

### 2.1. Germplasm Collection and Sample Preparation

**Figure 1:**
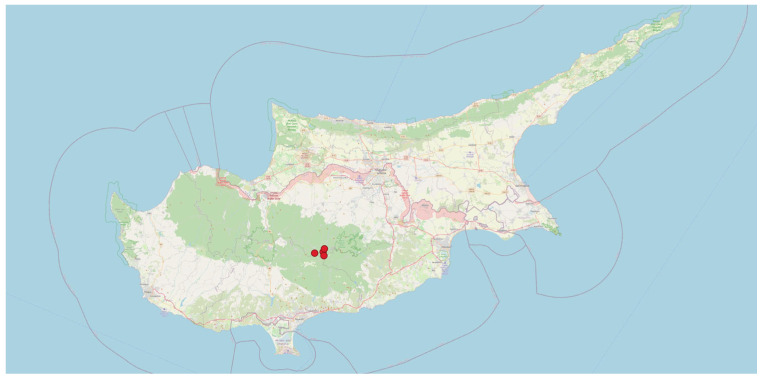
Collection sites of the Cypriot bean germplasm in the Troodos region. Precice coordinates are indicated with red dots. Base map © OpenStreetMap contributors (www.openstreetmap.org), licensed under ODbL.

Healthy young leaves from heirloom and commercial bean varieties were collected across Cypriot prefectures from farms in mountainous areas (Table 1; Figure 1). With the exception of variety “Moraleda”, all other genetic material is conserved by farmers (Farm Saved Seed), including the commercial variety “Stringless Blue Lake”. Except for “Moraleda” and “Stringless Blue Lake”, all other genetic material is exclusively cultivated in the Troodos massif, and it depicts high local adaptation. Samples were placed in zip bags and transferred to the laboratory using cool boxes. Tissues were frozen at −80 °C and subsequently freeze-dried for three days. Roughly 500 mg of dried material was pulverized (45″ at full speed) using a steel ball Mixer Mill MM 200 (Retsch, Haan, Germany). We established that 2 mL Eppendorf tubes (with rounded bases) and flash-freezing with liquid N_2_ before bead-beating had an improved efficiency in producing fine powder samples.

### 2.2. DNA Extraction

DNA was extracted using a modified CTAB (CTAB buffer: 2% Cetyl-Trimethyl-Ammonium-Bromide, 100 mM Tris-HCl, 1.4 M NaCl, 20 mM EDTA, 0.1% 2-mercaptoethanol, 1% polyvinyl pyrrolidone) procedure. For each freeze-dried, homogenized tissue (20 mg), 500 µL of CTAB extraction buffer (with 1 µL of 100 mM RNAse) was used. Samples were mixed/vortexed before transferring to a 60 °C water bath for 30 min and 10 min at room temperature. Following the incubation period, 500 µL of phenol–chloroform (1:1) was added, and samples were vortexed thoroughly before centrifugation for 5 min at 14,000× *g*. The aqueous upper phase was transferred to a new tube, and chloroform extraction (1 volume) followed. Samples were briefly vortexed before centrifugation (2 min at 14,000× *g*). The aqueous upper phase was transferred to a fresh tube, and DNA was precipitated by adding 1 volume of cold isopropanol. Following incubation (room temperature for 15 min), samples were centrifuged (14,000× *g* for 10 min). The supernatant was carefully decanted without disturbing the pellet and subsequently washed with 500 µL ice-cold 70% ethanol. Ethanol residuals were removed by centrifugation and drying for 2 min at 60 °C. The pellet was dissolved in 50 µL ddH_2_O, and DNA quality/quantity was assessed using 1% agarose gel electrophoresis and nanodrop spectrophotometry.

### 2.3. DArTseq Genotyping

**Figure 2:**
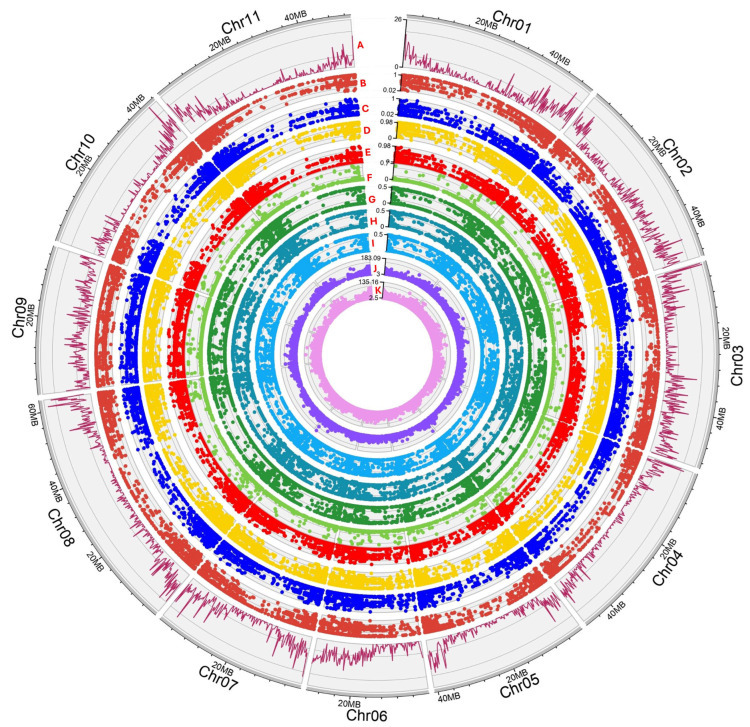
Circos depicting statistical indexes across the Cypriot germplasm collection for DArTseq markers. A. SNP density using a 200 Kbp bin rolling window; B. OneRatioRef: proportion of samples for which the genotype score is 0 (homozygous reference); C. OneRatioSnp: proportion of samples for which the genotype score is 2 (homozygous alternate); D. FreqHomRef: proportion of homozygous samples for the Reference allele; E. FreqHomSnp: proportion of homozygous samples for the Alternate (SNP) allele; F. FreqHets: proportion of samples which score as heterozygous, that is, scored as 1; G. PICRef: polymorphism information content (PIC) for the Reference allele; H. PICSnp: polymorphism information content (PIC) for the SNP; I. AvgPIC: average of the polymorphism information content (PIC) of the Reference and SNP alleles; J. AvgCountRef: sum of the tag read counts for all samples, divided by the number of samples with non-zero tag read counts, for the Reference allele row; K. AvgCountSnp: sum of the tag read counts for all samples, divided by the number of samples with non-zero tag read counts, for the Alternate (SNP) allele row.

DNA samples were sent to Diversity Arrays Technology Pty. Ltd. (Canberra, Australia) and genotyped using a ddRAD (double digest Restriction Associated DNA) genome complexity reduction method (DArTseq) as previously reported [43]. DArTseq libraries were constructed for the 50 genotypes using 50 ng of gDNA as a template. Briefly, DNA was digested using a combination of restriction enzymes (*Pst*I-*Mse*I), and generated fragments were ligated with enzyme-compatible adaptors before PCR amplification [44]. The DArTseq data were analyzed to produce dominant SilicoDArTs (presence or absence of the hybridized fragment) markers as well as SNPs. An e-value of 5 × 10^−7^ and a minimum sequence percent identity of 70% to a common bean genome reference (Common_bean_v12phy) were applied. Several statistical indexes were assessed and retained to establish the markers’ quality and information capacity: AvgCountRef: The sum of the tag read counts for all samples, divided by the number of samples with non-zero tag read counts, for the Reference allele row; AvgCountSnp: The sum of the tag read counts for all samples, divided by the number of samples with non-zero tag read counts, for the SNP allele row; AvgPIC: The average of the polymorphism information content (PIC) of the Reference and SNP allele rows; FreqHets: The proportion of samples which score as heterozygous; FreqHomRef: The proportion of samples which score as homozygous for the Reference allele; FreqHomSnp: The proportion of samples which score as homozygous for the SNP allele; OneRatioRef: The proportion of samples for which the genotype score is “1” in the Reference allele row; OneRatioSnp: The proportion of samples for which the genotype score is “1” in the SNP allele row; PICRef: The polymorphism information content (PIC) for the Reference allele row; PICSnp: The polymorphism information content (PIC) for the SNP allele row (Figure 2).

### 2.4. DArTseq Filtering and Analyses

**Figure 3:**
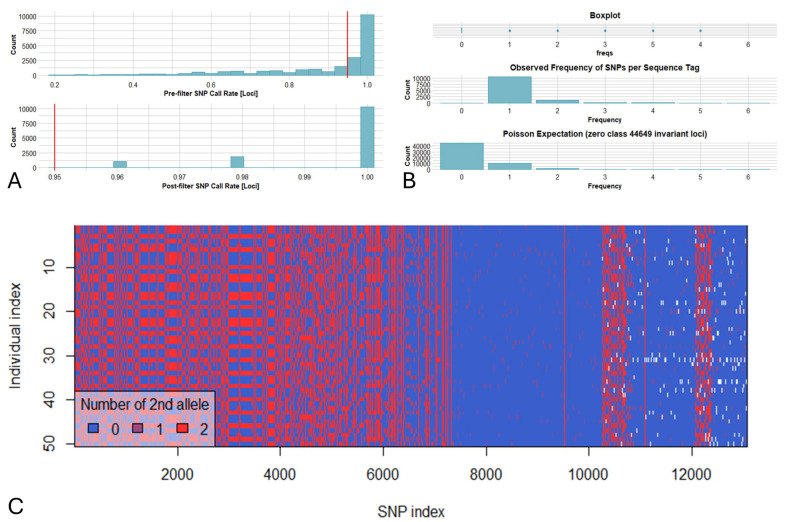
(**A**). Removing loci based on call rate (threshold = 0.95) resulted in the retention of 13,215 loci across 50 bean genotypes (eight populations). (**B**). Frequencies of SNPs detected across sequence tags depicting that a significant number of tags were polymorphic and that the majority of tags contained one SNP across the Cypriot bean germplasm. (**C**). Smear plot depicting color-coded loci by genotypes for DArTSeq scores of 0 (homozygous reference), 1 (heterozygous reference), 2 (homozygous alternate), and NA (null alleles).

Several concurrent cut-off values were used in order to trim data of lower quality. A call rate threshold of 0.95 (for loci) and 0.8 (for genotypes) was applied, while monomorphic loci were removed. This generated a matrix consisting of 50 genotypes and 13,215 retained loci, and a genlight object map (genotypes vs. SNPs) was created (Figure 3). The DArTseq statistic indexes across genotypes and loci alongside SNP density (bin window of 200 Kbp) were calculated (library “lattice”) across the 11 bean chromosomes, and a Circos plot graph was produced using shinyCircos-V2.0 [45]. Furthermore, several genetic attributes (private/fixed alleles per pair of populations, minor allele frequency (MAF) for each locus, diversity SNPs indexes, departure from Hardy–Weinberg equilibrium (HWE), and AMOVA) were calculated to characterize the Cypriot bean germplasm. DArTseq data were furthermore converted to PHYLIP/VCF formats, and phylogenetic/linkage disequilibrium (LD) analyses were performed using IQ-TREE [46] and Tassel 5 [47], respectively. To avoid a biased influence of the low frequency SNPs on LD, linkage disequilibrium (LD) was calculated using only SNPs with a MAF > 0.05. Linkage disequilibrium was assessed as the correlation of allele frequencies (r^2^) for every pairwise SNP comparison across each chromosome, and, thereafter, the mean values specific to the chromosome and genome were calculated. Inter-chromosomal linkage disequilibrium (unlinked loci) was assessed across the entire genome. The LD decay was assessed by creating a plot of the r^2^ values versus the genetic distance of loci pairs (Mbp) for each chromosome, and a trend line representing the LD decay was computed using locally weighted polynomial regression. All the above indexes were generated by using the DArTseq dedicated “dartRverse”/“dartR” R libraries and Rstudio (RStudio 2023.12.1 Build 402) as reported by the developers (https://green-striped-gecko.github.io/dartR/ (assessed on 19 September 2025)). Finally, a Bayesian statistic-based method for assessing genetic kinship was used employing the Structure 2.3.4 suite [48]. The admixture model was chosen, and five independent repeats for each K value (ranging from 1 to 8) were performed. Each run included 100,000 iterations of the burning period and 500,000 post-burn simulations as previously reported [35].

## 3. Results

### 3.1. SNP Indexes Across the Cypriot Bean Panel

The DArTseq analysis resulted in 21,111 silico DArT markers, as well as 10,260 SNP-containing sequence tags. In the present work, only SNPs were taken into consideration for the genetic analyses due to the non-binary/codominant nature of these markers. SNP markers presented an average call rate of 0.89 (before data trimming), and a low percentage (less than 5%) of null alleles. Selecting a minimum call rate of 0.95 resulted in a matrix of 13,215 SNPs, since various deviations from the reference genome were identified in several sequence tags (Figure 3). The mean reproducibility score was determined at nearly 99.9%, with an absolute value of 100% for most of the SNP markers (96%), while the lowest percentage was estimated at 88%.

The SNP markers were aligned to the 11 chromosomes of the bean reference genome, and an even genome-wide distribution was confirmed. The average SNP marker number per chromosome was 863.27 (±207.41), ranging from 588 in chromosome 4 to 1197 SNPs detected in chromosome 2 (Figure 2), corresponding to one SNP in every 40,651 Kbp (genome assembly of *P. vulgaris* v2.0 indicates a size of 537.2 Mbp, https://www.ncbi.nlm.nih.gov/datasets/genome/GCF_000499845.2/ assessed on 15 February 2025). A high density of markers was mainly observed at the chromosomal ends, even though chromosome 9 had a uniform marker dispersion and chromosome six presented a lower SNP density at the initial end (chromosome 6 is acrocentric). The reduced marker density in the rest of the chromosomes corresponded to their broader centromeric region, as expected. DArTSeq statistical indexes indicated that most genotypes were classified as homozygous to the *Phaseolus vulgaris* reference genome, since the FreqHomRef average value was 0.674. In contrast, FreqHomSnp had a mean score of 0.303, meaning that several genotypes were found to be homozygous to the alternative SNP. Interestingly, there were several cases where heterozygous genotypes were detected at a low (but significant) percentage (0.025), indicating a possible intercrossing within the Cypriot beans’ germplasm. SNP markers were proven to be highly informative, having an average Polymorphism Information Content (PIC) value of 0.295 (Figure 2).

### 3.2. Linkage Disequilibrium Across Markers

**Figure 4:**
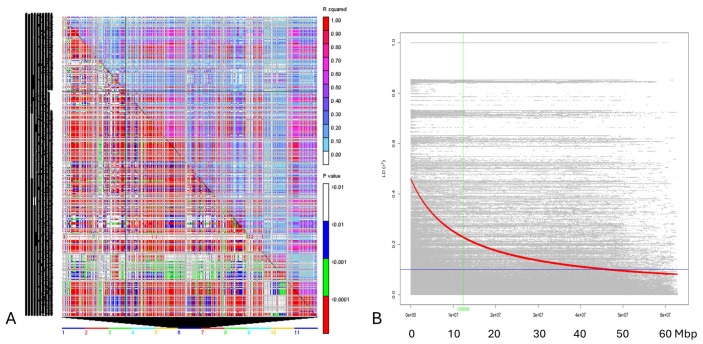
(**A**). Details of pairwise LD values of polymorphic sites are plotted on both the X and Y axes in the triangle plot created by TASSEL for pairwise LD between SNP sites across the 11 bean chromosomes (rolling window 500). The corresponding *p*-values from the rapid 1000 shuffle permutation test are shown below the diagonal, while r^2^ values are displayed above. Using color codes to indicate the existence of substantial LD, each cell compares two pairs of marker sites. Threshold values on both diagonals are indicated by colored bar codes. (**B**). Scatter plot showing the linkage disequilibrium (LD) decay across the genome of the Cypriot bean germplasm. The genetic distance in bp is plotted against the LD estimate (r^2^) for each pair of SNP markers. The red line represents the trend of non-linear regressions against physical distance. The horizontal and vertical lines indicate the crucial values for r^2^ (0.2) and LD decay levels, respectively.

Linkage disequilibrium estimates (as r^2^) and the degree of LD decay were determined among the 50 Cypriot accessions by using all possible pair combinations of genome-wide physically mapped SNP markers on the 11 bean chromosomes. A significant LD was observed for several SNP pairs within the germplasm (*p* < 0.001), and r^2^ values higher than 0.90 were observed at several sites (Figure 4A) within the chromosomes. Nonetheless, many loci had r^2^ values less than 0.5, proving that LD is not heavily widespread in the Cypriot bean landraces. Specifically, average r^2^ values across the 11 chromosomes varied from 0.225 (chromosome 5) to 0.423 (chromosome 9), while 25.56% (ranging from 21.46% in chromosome 5 to 28.93% in chromosome 4) of marker-pairs demonstrated significant a LD score (*p* < 0.001). Hence, moderate LD levels in the panel of Cypriot beans were confirmed. Significant discrepancies among linked (*p* < 0.001) and unlinked (*p* > 0.001) markers were also noted, since r^2^ values in unlinked SNP markers were below the 0.1 threshold across chromosomes (0.78 on average, ranging from 0.064 in chromosome 1 to 0.093 in chromosome 9). Moreover, the extent of LD decay was calculated using a non-linear regression of r^2^ values versus the physical chromosomal distance in the 50 Cypriot bean panel (Figure 4B). Linkage disequilibrium followed a gradual declining trend as physical distance increased, even though a sharp drop within the first 10 Mbp was not established. The germplasm studied had intermediate LD values, and most r^2^ records (correlations among locus pairs) were below the 0.5 threshold, while bean genotypes sustained a significant LD level up to a physical distance of 20 Mbp.

### 3.3. Alleles, HWE, and Genetic Diversity

**Figure 5:**
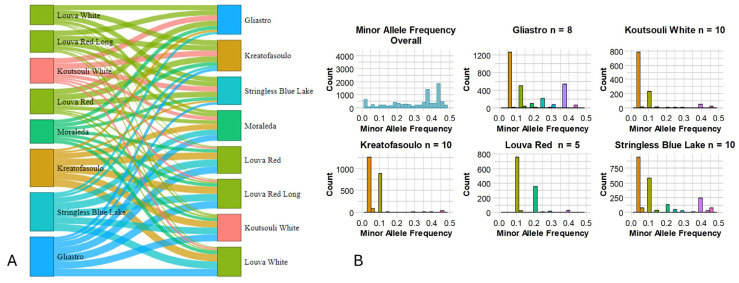
(**A**). A Sankey Diagram was utilized to display patterns of private alleles across populations and flows (private alleles) between nodes (varieties). Their connections are depicted by arcs with widths proportionate to the relevance of the flow (number of private alleles). (**B**). Minor allele frequency across the Cypriot bean germplasm.

A survey for private and fixed alleles across varietal combinations was performed (Table 2) and a Sankey Diagram depicting the complex relationships among genotypes was produced (Figure 5A). The minimum number of fixed alleles was estimated between the “Louva White” and “Louva Red” varieties (6 alleles), while the maximum was found among “Kreatofasoulo” and “Louva White” genotypes (5165 alleles). Since low genetic diversity is often linked to allele fixation, the moderate values detected for Cypriot beans indicate a possible decreased adaptability for some genotypes. “Gliastro” was also characterized by many private alleles (when compared with other cultivars) since, on average, approximately 5920 unique alleles were recorded. Interestingly, “Louva” types had a small number of private alleles when compared in pairs (Figure 5A), indicating that, regardless of the color (red or white) or pod length (normal or long), these heirloom varieties have significant genetic affiliations. Besides exploring the private/fixed alleles among varieties, a search for alleles having a minor frequency (less than 0.5%) was conducted, and the MAF (minor allele frequency) histogram (Figure 5B) revealed several fixed variants (SNPs) in the Cypriot common bean genome. It was established that, overall, the majority of the MAF was evenly distributed across the 0.01% and 0.5% threshold (mean 0.217%; median 0.2%; first quantile 0.010%; third quantile 0.380%) although in several varieties (“Gliastro”, “Koutsouli White”, “Kreatofasoulo”, and “Stringless Blue Lake”) most minor alleles had a frequency less than 0.2%.

**Figure 6:**
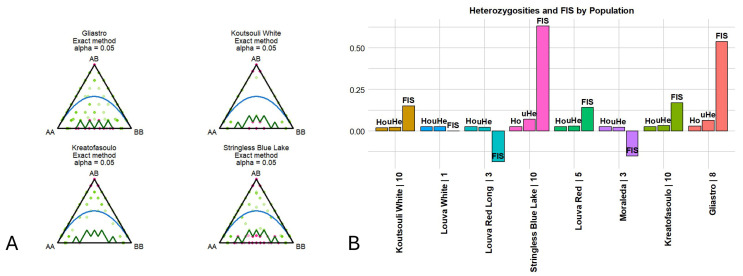
(**A**). Significant departures of HWE. HW equilibrium agreement was based on observed frequencies of reference homozygotes, heterozygotes, and alternate homozygotes as previously described [49]. (**B**). Indexes of heterozygosity and inbreeding across the Cypriot bean germplasm as calculated across 13,078 SNP loci. Observed heterozygosity (Ho) = number of homozygotes/n_Ind, where n_Ind is the number of genotypes without missing data. Expected heterozygosity (He) = 1 − (*p*^2^ + *q*^2^), where *p* is the frequency of the Reference allele and q is the frequency of the Alternative allele. Unbiased expected heterozygosity (uHe) = He × (2 × n_Ind/(2 × n_Ind − 1)). Inbreeding coefficient (FIS) = 1 − (mean(Ho)/mean(uHe)).

Concurrently, a test for Hardy–Weinberg disequilibrium was conducted to detect loci where a nonrandom assortment of alleles occurred. Interestingly, across the loci and varieties studied, we identified 10 loci under Hardy–Weinberg disequilibrium (Figure 6A; Table 3). “Stringless Blue Lake” was the variety where most departures were noted (six), followed by “Gliastro” (two), while for “Koutsouli White” and “Kreatofasoulo” only one locus was found in HW disequilibrium. The loci identified were further screened against the NCBI nr database using blastn (https://blast.ncbi.nlm.nih.gov/Blast.cgi, assessed on 2 March 2025) in order to identify possible functions. Seven (out of the ten loci) corresponded to disease-resistant genes (XM_068642443.1, XM_068615419.1, XM_068628962.1) or crucial enzymes implicated in the primary or secondary metabolism (Table 3).

Across the 13,078 loci retained for the analyses, approximately 12% were found to be polymorphic. Moreover, low levels of observed (Ho), expected (He), and unbiased heterozygosity (uHe) were detected across populations (Figure 6B; Table 4). “Koutsouli white” and “Louva Red Long” were the varieties where the lowest values of heterozygosity were detected (Ho = 0.018/uHe = 0.021 and Ho = 0.025/uHe = 0.021, respectively), while the highest rates were determined for “Gliastro” and “Stringless Blue Lake” (Ho = 0.030/uHe = 0.065 and Ho = 0.025/uHe = 0.069, respectively). Moreover, inbreeding coefficient analysis (FIS) indicated that “Stringless Blue Lake” and “Gliastro” varieties had a high probability of inbreeding (FIS = 0.634 and FIS = 0.540, correspondingly), while negative scores were determined for “Louva Red Long” and “Moraleda” (FIS = −0.187 and FIS = −0.152, respectively), signifying an evasion of inbreeding. Most varieties, however, had near zero values, suggesting that the degree of inbreeding was equal to the expected value based on allelic rates of the germplasm.

### 3.4. Varieties Associations and Genetic Structure

**Figure 7:**
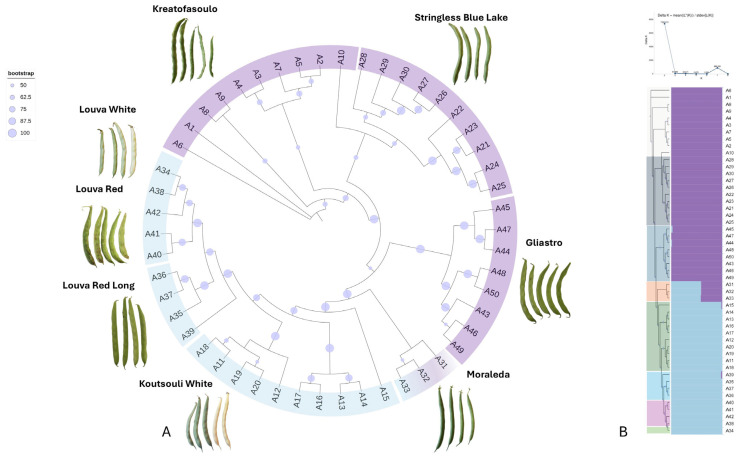
(**A**) Phylogenetic dendrogram based on DArTseq-derived SNP data, constructed using the approximate likelihood-ratio test (aLRT) method. Bootstrap support values are indicated as circles at each node, reflecting the confidence level of branching. Genotypes cluster primarily according to usage type (dry vs. green beans). (**B**) STRUCTURE bar plot representing genetic clustering (K = 2) across 50 Cypriot *Phaseolus vulgaris* genotypes. Each vertical bar represents one genotype, and colors indicate membership proportions to inferred genetic clusters. A clear separation between green and dry bean types is visible, with admixture observed in select varieties such as “Moraleda” and “Gliastro”.

Genetic affiliations among the germplasm were calculated by using an approximate likelihood-ratio test (aLRT) branch algorithm, and a dendrogram with bootstrap support was produced. Genotypes of the same variety were generally grouped within the same cluster, and these associations were further supported by high branch probability values (Figure 7A). Louva types (“Louva White”, “Louva Red”, and “Louva Long Red”) formed a separate cluster that had “Koutsouli White” as an outgroup. Interestingly, most of these genotypes have a dry bean use, signifying that genetic kinship corresponds to selected agronomical traits. “Gliastro”, “Stringless Blue Lake”, and “Kreatofasoulo” also exhibited high genetic affiliations that corresponded to their use (green beans), as these genotypes formed a second larger cluster. Interestingly, “Stringless Blue Lake” genotypes formed two discrete subgroups depending on their origin (farmers’ site), since these constitute genetic resources that are conserved “on-farm”. Despite the fact that “Moraleda” is classified as a green bean variety, it had an intermediate genetic markup, as its genotypes had an intermediate classification.

A Bayesian structure analysis was additionally performed to survey the distribution of genetic clusters and the admixture within the bean germplasm. The optimal score for the ad hoc test, based on the second-order rate of change in the probability function regarding the deltaK index, was determined for K = 2 (ΔK = 7395). In general, bean genotypes were rather genetically homogeneous, although several cases of admixture were noted (Figure 7B). Specifically, the majority of green bean genotypes were confidently clustered in the first genetic group (lilac color), with the exception of the “Moraleda” genotypes that were admixtured; on average, we observed a 0.42 similarity to cluster 1 and a 0.48 identity to cluster 2 (cyan color). On the contrary, dry bean genotypes were clustered to the second genetic group (Figure 7B). Minor admixtures (less than 0.02) were also noted for genotypes of “Koutsouli White” (one genotype), “Louva Red” (one genotype), and “Gliastro” (two genotypes), signifying that genetic flow may exist among different varieties during pollination. Finally, the analysis of molecular variance (AMOVA) determined that significant variation exists between varieties and between samples within varieties, but a significant within-sample variation was not revealed (Supplementary Figure S1), suggesting that population structure occurs.

**Figure 8:**
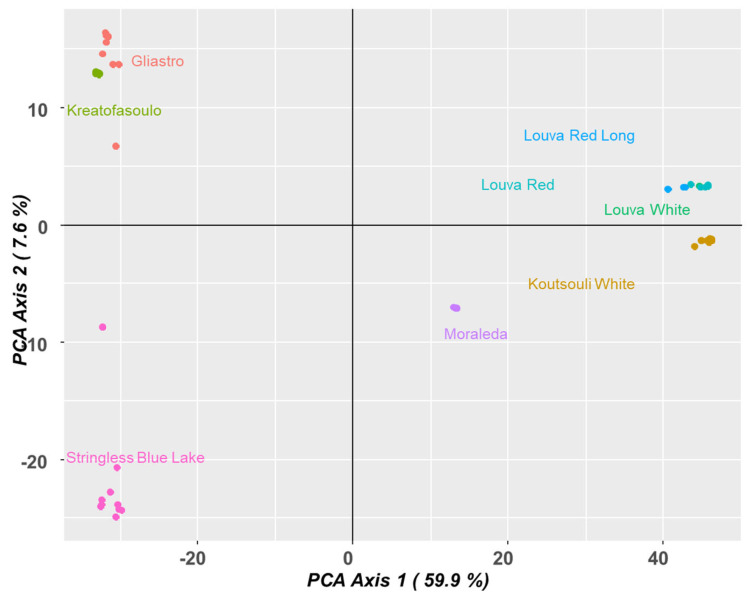
PCA analysis depicting the genetic associations across Cypriot bean genotypes.

Finally, a PCA plot analysis was conducted (Figure 8). The PCA plot of the Phaseolus genotypes also demonstrated that there are differences between genetic backgrounds within the groups. It appears that the first axis (59.9% of variation) was the main axis in differentiating the other genotypes. On the far right of the PCA plot, the clustering of “Louva” genotypes suggests a close genetic similarity, and “Koutsouli White” is positioned in a way that suggests it is somewhat related to their genetic background. On the left side of the PCA plot, “Gliastro” and “Kreatofasoulo” both fall into the upper quadrant and represent a different cluster than the “Louva” genotypes. There is significant overlap in the middle of the plot with “Moraleda”, as it is positioned between “Louva” and “Stringless Blue Lake” in the lower left quadrant, and yet the positioning suggests some evidence of a distinction between them. “Stringless Blue Lake” falls just below the upper left quadrant, and, despite being close to the other genotypes, it also appears to be somewhat isolated, representing a distant cluster.

## 4. Discussion

This work presents the first comprehensive DArTseq-based genetic analysis of the Cypriot common bean (*Phaseolus vulgaris* L.) gene pool that aims to study the unexploited genetic variation within heirloom dry and green bean genotypes originating from remote mountainous areas. Supported by a high-density SNP dataset that contained 13,215 markers, we were able to confirm the unique status of Cypriot beans and provide evidence of their possible value for conservation as well as breeding programs.

The common bean was brought to Europe shortly after the Columbian Exchange, when Spanish and Portuguese conquistadors planted Andean and Mesoamerican landraces in the Old World during the 16th century [3,5]. These original introductions from each gene pool presumably underwent a number of rounds of dissemination and local selection throughout Europe. As a result, European landraces, especially in the south, such as the Mediterranean, formed unique genotypic groups due to ecological adaptation, farmer preference, and seed exchange behavior [6,7], creating also the base for heirloom varieties. Landraces and traditional cultivars tend to have a higher genetic diversity compared to modern cultivars due to their broader allelic base and minimal exposure to intensive breeding bottlenecks. This, in fact, was noted in the current study (Cypriot panel), wherein heirloom varieties such as “Gliastro” and “Kreatofasoulo” exhibited high frequencies of private alleles and polymorphic loci. This is in accordance with genetic resources in Ethiopia [29] and Turkey [32], wherein landraces were reported to possess a higher intra-population diversity compared to elite lines. The low values of observed heterozygosity (0.018 to 0.030 for Ho) in the Cypriot bean collection are in line with the self-pollinating nature of *P. vulgaris*, but the prevalence of high minor allele frequencies and a Hardy–Weinberg disequilibrium in certain loci indicates that genetic diversity is still preserved in the traditional cultivars.

Moreover, the observed average PIC value (0.295) is in correlation with other reports utilizing the DArTseq in beans. For example, Gelaw et al. [29] noted a mean PIC value of 0.30 in the Ethiopian germplasm (the study involved 11,480 SNPs), while Wahome and colleagues [27] reported slightly lower values (0.27) in 222 Kenyan genotypes. These similarities attest that the Cypriot germplasm has moderate to high levels of polymorphism, despite being geographically isolated. The very high reproducibility (mean 99.9%) and extensive coverage of the genome confirm the validity of the marker system employed. Linkage disequilibrium (LD) analysis indicated that there were relatively moderate levels of LD across the Cypriot panel, from 0.225 (Chromosome 5) to 0.423 (Chromosome 9). These are at the range obtained for Ethiopian genotypes (mean r^2^~0.35) [29], but are in contrast to the steeper LD decay in more bottlenecked populations such as those in Turkey [32,50], where LD was observed in larger distances. The Cypriot panel showed a more gradual decline in LD without an abrupt drop in the first 10 Mbp, which might reflect a previous recombination and a low selection pressure, possibly due to conventional seed exchange and in situ conservation practices.

Structure analysis (STRUCTURE and phylogenetic clustering) identified two major genetic groups based mostly on usage type: “Koutsouli White”, “Stringless Blue Lake”, “Moraleda”, “Louva White”, “Louva Red”; and “Gliastro”, “Kreatofasoulo”, “Koutsouli White”, “Stringless Blue Lake”, and “Moraleda”. This division suggests simultaneous domestication routes or divergence post-introduction through utilization and selection. A similar divergence has been earlier documented by Wahome et al. [27], who reported clustering by agronomic use lines. Despite this dichotomy, admixture was detected—predominantly in “Moraleda” and also in some “Gliastro” genotypes—reflecting an ongoing gene flow between bean varieties in Cyprus, which is highly likely to be an artifact of traditional farming practices. Notably, the “Stringless Blue Lake” group, which clusters into two sub-clusters by farm origin, is an illustration of the study by Correa Abondano et al. [26], where they demonstrated how sampling strategy may reveal micro-structure within landrace groups. This supports the factoring in of farm-level management and local selection when interpreting bean genetic diversity.

As opposed to the greater differentiation between the Mesoamerican and Andean gene pools in other regions, the Cypriot genotypes are less stratified and more admixed. This may be due to centuries of Mediterranean germplasm flow and farmers’ seed exchange, which diluted the dichotomy of the gene pools. The relatively low observed heterozygosity agrees with expectations in self-pollinated crops and is in agreement with results from other studies in European landraces [30]. Even at low values of Ho, signs of private alleles (particularly in “Gliastro”) and Hardy–Weinberg disequilibrium loci (particularly in “Stringless Blue Lake”) reveal a hidden variability and potential selection of gene flow events. The detection of disease-resistance and stress-response loci among HWE-violating SNPs echoes reports by [27], who found high GWAS associations within corresponding genomic regions. These findings show the functional promise of the Cypriot bean genome for adaptive traits.

Finally, of particular interest, DArTseq use possessed an adequate resolution to distinguish highly similar landraces like “Louva Red” and “Louva White,” which were distinguished by a six-allele difference, while unrelated types exceeded 5000. Such resolution explains the usefulness of DArTseq in defining cohesion-by-descent relationships and guiding conservation strategies, as also emphasized by Correa Abondano and colleagues [26].

## 5. Conclusions

This study provided a valuable Mediterranean dataset to the global *P. vulgaris* DArTseq literature. It highlighted Cyprus as a secondary hotspot of diversity with a unique, admixed germplasm that is of high priority for in situ and ex situ conservation. The genetic differentiation that is apparent between dry and green beans, the richness of landraces’ diversity, and the traces of former admixture further highlight the importance of conserving these genetic materials. Future work should combine phenotypic and metabolomic information in order to better determine the adaptive worth and function of these landraces.

## Figures and Tables

**Table 1:** Cypriot *P. vulgaris* accessions analyzed in this study, and their classification by origin and usage type.

No	Variety Name	Origin	Type
1	Kreatofasoulo	Heirloom variety collected from field	Green beans
2	Kreatofasoulo	Heirloom variety collected from field	Green beans
3	Kreatofasoulo	Heirloom variety collected from field	Green beans
4	Kreatofasoulo	Heirloom variety collected from field	Green beans
5	Kreatofasoulo	Heirloom variety collected from field	Green beans
6	Kreatofasoulo	Heirloom variety collected from field	Green beans
7	Kreatofasoulo	Heirloom variety collected from field	Green beans
8	Kreatofasoulo	Heirloom variety collected from field	Green beans
9	Kreatofasoulo	Heirloom variety collected from field	Green beans
10	Kreatofasoulo	Heirloom variety collected from field	Green beans
11	Koutsouli White	Heirloom variety collected from field	Dry beans
12	Koutsouli White	Heirloom variety collected from field	Dry beans
13	Koutsouli White	Heirloom variety collected from field	Dry beans
14	Koutsouli White	Heirloom variety collected from field	Dry beans
15	Koutsouli White	Heirloom variety collected from field	Dry beans
16	Koutsouli White	Heirloom variety collected from field	Dry beans
17	Koutsouli White	Heirloom variety collected from field	Dry beans
18	Koutsouli White	Heirloom variety collected from field	Dry beans
19	Koutsouli White	Heirloom variety collected from field	Dry beans
20	Koutsouli White	Heirloom variety collected from field	Dry beans
21	Stringless Blue Lake	Variety from farmer A collected from field	Green beans
22	Stringless Blue Lake	Variety from farmer A collected from field	Green beans
23	Stringless Blue Lake	Variety from farmer A collected from field	Green beans
24	Stringless Blue Lake	Variety from farmer A collected from field	Green beans
25	Stringless Blue Lake	Variety from farmer A collected from field	Green beans
26	Stringless Blue Lake	Variety from farmer B collected from field	Green beans
27	Stringless Blue Lake	Variety from farmer B collected from field	Green beans
28	Stringless Blue Lake	Variety from farmer B collected from field	Green beans
29	Stringless Blue Lake	Variety from farmer B collected from field	Green beans
30	Stringless Blue Lake	Variety from farmer B collected from field	Green beans
31	Moraleda	Variety from nursery collected from field	Green beans
32	Moraleda	Variety from nursery collected from field	Green beans
33	Moraleda	Variety from nursery collected from field	Green beans
34	Louva White	Heirloom variety collected from field	Dry beans
35	Louva Red Long	Heirloom variety collected from field	Dry beans
36	Louva Red Long	Heirloom variety collected from field	Dry beans
37	Louva Red Long	Heirloom variety collected from field	Dry beans
38	Louva Red	Heirloom variety collected from field	Dry beans
39	Louva Red	Heirloom variety collected from field	Dry beans
40	Louva Red	Heirloom variety collected from field	Dry beans
41	Louva Red	Heirloom variety collected from field	Dry beans
42	Louva Red	Heirloom variety collected from field	Dry beans
43	Gliastro	Heirloom variety collected from field	Dry beans
44	Gliastro	Heirloom variety collected from field	Dry beans
45	Gliastro	Heirloom variety collected from field	Dry beans
46	Gliastro	Heirloom variety collected from field	Dry beans
47	Gliastro	Heirloom variety collected from field	Dry beans
48	Gliastro	Heirloom variety collected from field	Dry beans
49	Gliastro	Heirloom variety collected from field	Dry beans
50	Gliastro	Heirloom variety collected from field	Dry beans

**Table 2:** Pairwise comparisons of fixed/private alleles among the Cypriot bean collection. Allele frequency differences (AFD) and diversity estimates (Chao1/Chao2) illustrate genetic differentiation and distinctiveness among variety pairs.

No	Variety 1	Variety 2	Fixed	Priv1	Priv2	Chao1	Chao2	Totalpriv	AFD
1	Gliastro	Koutsouli White	4435	7191	5545	714	220	12,736	0.456
2	Gliastro	Kreatofasoulo	472	2858	2357	714	829	5215	0.115
3	Gliastro	Louva White	4921	7824	5159	714	43	12,983	0.467
4	Gliastro	Louva Red	4638	7453	5809	714	473	13,262	0.466
5	Gliastro	Louva Red Long	4793	7677	5348	714	412	13,025	0.459
6	Gliastro	Moraleda	3151	5982	3736	714	300	9718	0.323
7	Gliastro	Stringless Blue Lake	413	2453	2494	713	541	4947	0.144
8	Koutsouli White	Kreatofasoulo	4628	5788	6933	220	829	12,721	0.44
9	Koutsouli White	Louva White	772	1970	951	220	43	2921	0.083
10	Koutsouli White	Louva Red	626	1728	1730	220	473	3458	0.083
11	Koutsouli White	Louva Red Long	950	2124	1441	220	412	3565	0.099
12	Koutsouli White	Moraleda	2435	3634	3034	220	299	6668	0.229
13	Koutsouli White	Stringless Blue Lake	4170	5267	6954	220	541	12,221	0.443
14	Kreatofasoulo	Louva White	5165	7586	5422	829	43	13,008	0.456
15	Kreatofasoulo	Louva Red	4896	7225	6082	829	473	13,307	0.455
16	Kreatofasoulo	Louva Red Long	5026	7418	5590	829	412	13,008	0.448
17	Kreatofasoulo	Moraleda	3391	5722	3977	829	300	9699	0.318
18	Kreatofasoulo	Stringless Blue Lake	389	2011	2553	828	541	4564	0.123
23	Louva Red	Louva Red Long	390	1547	862	473	412	2409	0.053
24	Louva Red	Moraleda	2770	3978	3376	473	299	7354	0.247
25	Louva Red	Stringless Blue Lake	4497	5665	7350	473	541	13,015	0.463
26	Louva Red Long	Moraleda	2861	3395	3478	412	300	6873	0.243
27	Louva Red Long	Stringless Blue Lake	4615	5158	7528	412	541	12,686	0.456
19	Louva White	Louva Red	6	145	1166	42	473	1311	0.019
20	Louva White	Louva Red Long	457	626	962	43	412	1588	0.05
21	Louva White	Moraleda	2925	3157	3576	43	300	6733	0.246
22	Louva White	Stringless Blue Lake	4754	4993	7699	43	541	12,692	0.463
28	Moraleda	Stringless Blue Lake	2876	3435	5722	299	541	9157	0.314

**Table 3:** Loci exhibiting significant deviations from Hardy–Weinberg equilibrium across the selected genotypes. Loci identified and their functional annotation from NCBI BLAST highlight the potential association to disease resistance and metabolic processes, mainly in cultivars.

Variety	Locus	Hom_1	Het	Hom_2	N	Prob	NCBI Accession	e Value
Gliastro	100062028-27-C/T	2	0	6	8	0.01538	-	-
Koutsouli White	100068280-6-T/C	0	10	0	10	0.00691	XM_068642443.1 ^1^	9.00 × 10^−21^
Stringless Blue Lake	13121316-28-G/A	5	0	5	10	0.00136	XM_068640160.12 ^2^	0.002
Stringless Blue Lake	13121337-48-C/T	5	0	5	10	0.00136	-	-
Stringless Blue Lake	13121391-56-C/A	4	1	4	9	0.03645	XM_068606144.1 ^3^	7.00 × 10^−29^
Stringless Blue Lake	8671140-23-T/C	5	0	5	10	0.00136	XM_068641491.1 ^4^	2.00 × 10^−9^
Stringless Blue Lake	8671150-35-T/C	1	9	0	10	0.04549	XM_068641720.1 ^5^	6.00 × 10^−22^
Stringless Blue Lake	8671153-41-A/C	7	1	2	10	0.04644	XM_068615419.1 ^6^	8.00 × 10^−19^
Kreatofasoulo	8671195-24-A/C	0	9	1	10	0.04549	-	-
Gliastro	8671212-10-T/C	3	0	5	8	0.00699	XM_068628962.1 ^7^	0.005

^1^: *Phaseolus vulgaris* probable disease resistance protein At4g27220 (LOC137834386), mRNA; ^2^: *Phaseolus vulgaris* SNF1-related protein kinase regulatory subunit beta-2-like (LOC137832141), mRNA; ^3^: *Phaseolus vulgaris* small ribosomal subunit protein uS5w-like (LOC137806161), mRNA; ^4^: *Phaseolus vulgaris* probable N-acetyltransferase HLS1-like (LOC137833118), mRNA; ^5^: *Phaseolus vulgaris* non-specific lipid-transfer protein AP10-like (LOC137833370), mRNA; ^6^: *Phaseolus vulgaris* rust resistance kinase Lr10-like (LOC137813293), mRNA; ^7^: *Phaseolus vulgaris* putative disease resistance RPP13-like protein 1 (LOC137823721), transcript variant X1, mRNA.

**Table 4:** Genetic diversity indexes per bean cluster based on DArTseq-derived SNPs. Variables (Ho, He/uHe, and FIS) reflect the elevated intra-varietal genetic diversity.

Variety	polyLoc	monoLoc	Ho	He	uHe	FIS
Koutsouli White	1331	11,747	0.018	0.020	0.021	0.151
Gliastro	2977	10,101	0.030	0.060	0.065	0.540
Stringless Blue Lake	3018	10,060	0.025	0.066	0.069	0.634
Louva Red	1333	11,745	0.026	0.027	0.030	0.141
Kreatofasoulo	2476	10,602	0.027	0.031	0.032	0.168
Louva Red Long	648	12,430	0.025	0.017	0.021	−0.187
Moraleda	731	12,347	0.027	0.019	0.023	−0.152
Louva White	312	12,766	0.024	0.012	0.024	0.000

## Data Availability

The raw data supporting the conclusions of this article will be made available by the authors on request.

## Supplementary Materials

The following supporting information can be downloaded at https://www.mdpi.com/article/10.3390/plants14193000/s1. Figure S1: AMOVA plots depicting the distribution of the variance components (using permutation tests) at three hierarchical levels: (top) among samples, (middle) among samples, and (bottom) among populations. Black diamonds represent the estimated variance component for each level. Sizeable deviations of the observed values from the null distribution reflect hierarchical genetic structure at the given level.
