# Supplementary figures and images for: DArTseq Analysis of Cypriot Common Bean Germplasm Unveils an Assortment of Unexplored Genetic Variability
Source: Plants (Basel). 2025 Sep 28;14(19):3000. doi: 10.3390/plants14193000 (PMC12525803; doi:10.3390/plants14193000)

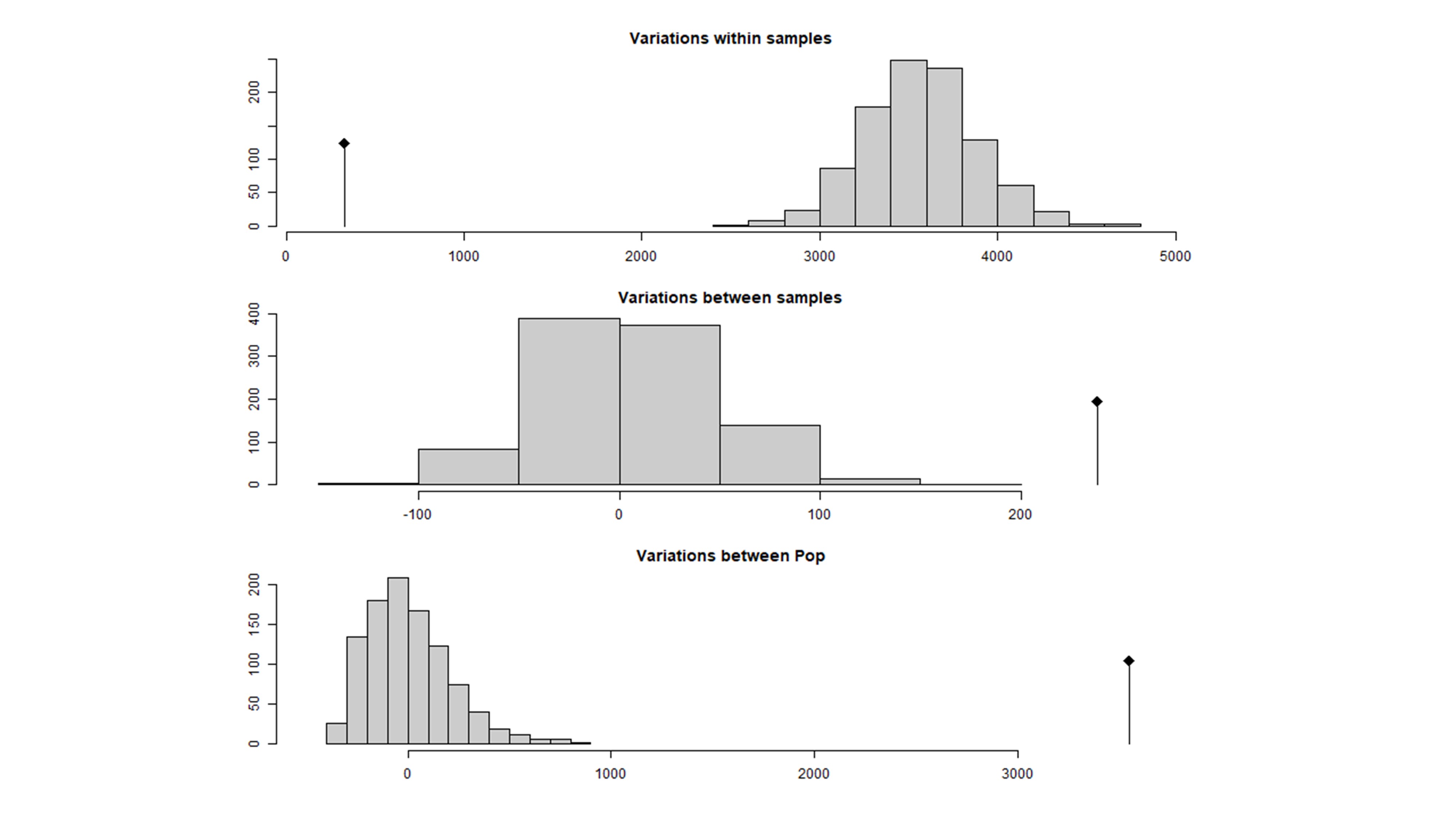

Supplement: Supplementary file 1 [file plants-14-03000-s001.zip › plants-3873349-supplementary.JPG]
